# Lecture on Secale Cornutum

**Published:** 1885-01

**Authors:** J. T. Kent

**Affiliations:** St. Louis


### LECTURE ON SECALE CORNUTUM.

Professor J. T. Kent, M. D., St. Louis.

The Ergot is a drug that acts very powerfully on the human system.

When given in very large doses, sufficiently to impress the whole system with its power, one of the first influences wrought is a peculiar constringent feeling throughout the whole body.

You will not practice surgery long before you will discover a demand for Ergot in cases of aneurisms ; then you will have an opportunity of making a partial proving of it in large doses. But your patient will come back after taking a drachm of the fluid extract of Ergot for surgical purposes, telling you that he feels as if the muscular fibres of his body were contracted ; his eyes seem to be pulling ; the unstriped fibres of the body undergo spasmodic action or contraction ; if carried on to great violence, would amount to spasms ; if carried only to a moderate degree of contraction, it simply produces a peculiar drawing effect that is felt in various parts of the body.

The first effect noticed is upon the brain—the blood-vessels of the brain—because, perhaps, this is the most sensitive part of the body ; next upon the uterus.

Of course, we can't say but that the same is true of all the unstriped fibres throughout the body, that they are all acted on in this way, but there is a more sensitive condition in the brain, and hence the more noticeable effect.

Now, if you have already an irritated state, that irritable part will be sought out first for action ; hence we see in the gravid uterus, which is thoroughly prepared for the action of Ergot by virtue of this peculiar irritability, this state. Now, Secale in this case will act primarily on the uterus. In the state of parturition large doses will act primarily upon the uterus, and the brain will seem to entirely escape. But in the non-gravid uterus, or in the male, for instance, in an irritable brain, you will find that Secale will seek out the cause of the irritability, the brain first, primarily, and will exhaust itself.

Now, primarily, what does Ergot do on the venules and arterioles, in all parts of the body ? Simply to contract the calibre, producing anemia ; hence it is that the old school physician resorts to large doses of Secale in hyperæmia. If he has a congestion of the brain, or an irritation of the brain attended with dilated blood-vessels, dilated arterioles, he will resort to Ergot to contract those blood-vessels, to force them within their proper calibre, and thereby affecting, primarily, the circulation of the blood. But whether the blood-vessel was dilated or not, Ergot will contract the calibre of the blood-vessel when used in large doses. It will do that same thing in the circular organs, I mean in the cavernous organs. It will do it in the uterus, also in the heart and blood-vessels.

This comes from the early effect of the drug.

Although we have gone through a review of this primary effect, it is of no value to the homœopathist.

Now we pass to the secondary effect, which is the result of anemia, and from which we get the symptoms that guide us particularly to the homœopathic use. The secondary effect of the drug is that of anemia.

The parts become shriveled, and particularly in the extremities. The parts are numb, particularly in the extremities. The hands and feet become shriveled and bluish; become dusky, mottled, and petecchiæ form. We have tingling and numbness of the hands and feet.

And finally, if this goes on, as it has done in the Indian-bread eaters, we find gangrenous states very similar to senile gangrene of old people. Hence it has been useful in the shriveled condition of the limbs and the emaciation belonging to old people.

So there is anemia of the brain and childishness; and the shriveled condition of the skin—the skin appearing as if the hand had been plunged a long time in hot water, then withdrawn and dried, the shriveling remaining; then it becomes dusky, and is covered with petecchiæ.

These are among the very marked features as the result of anemia. The gangrenous states are those which come on from anemia—a gangrenous state that is dry. Secale doesn't correspond to the rapid gangrene which you will find under Lachesis and peculiar to other remedies; it comes on slower from a gradual closure of the blood-vessels of the affected parts.

There is another general anemic condition of the body that is very peculiar. Secale is grandly in contrast with a remedy that is similar to it in every other way—Arsenicum.

Secale is grandly in contrast with that remedy. Why?

Because all of these symptoms and states are better from cold in Secale.

And all these conditions are better by heat in Arsenic patients.

In Secale we have great prostration and anxiety and thirst, and these are all peculiar to Arsenicum; great restlessness, anxiety, and thirst.

We have unquenchable thirst, as in Arsenicum ; no satisfaction from drinking.

There are many other states in harmony with Arsenicum that I shall mention as we go.

Secale produces another grand general effect, and that is a weakness of the coatings of blood-vessels; relaxation of the coatings of the blood-vessels.

When this drug is taken in great quantities and then stopped suddenly, you get a peculiar and marked effect, that is, the relaxation of the blood-vessels, and oozing from the capillaries.

There is hemorrhage from the nose, hemorrhage from the mouth of an oozing character, oozing from the gums, vomiting of blood, oozing from the bowels and the rectum, uterine hemorrhage, and hemorrhage from the kidneys, all of a capillary character. This hemorrhage is the secondary action of Ergot, and is the oozing of uncoagulated thin, dark, and black blood. You seldom have the clots in Ergot that you have in Belladonna and in Sabina and in Ipecac. Fluids are thin and watery.

It is in this secondary state of Ergot that we find the most characteristic peculiarities and the main strength of the remedy.

It produces upon the body, as a part of this peculiarity, destruction of the red corpuscles, and in that way producing another form of anemia.

The contraction of calibres produces only local anemia; the part acted upon will become anemic, but after a little while this destruction of blood corpuscles begins, and we have a general anemia.

This goes on in a progressive way until a gangrenous state sets in; petecchiæ and blisters form upon the body that are filled with thin fluid—serum—as we have in Lachesis, and something like we have in Cantharis.

We have now, in a general way, a modification of all the inflammatory states and irritability of the body. There is a general loss of irritability. But there are some features of marked irritability that appear like an inflammatory state because of the passive burning.

Now, there is burning in cavities. There is burning in the tissues. There is burning in the gangrenous state. There is burning in the abdomen. There is burning all throughout this remedy, and is characteristic, as in Arsenicum. But the burning of Arsenicum is ameliorated by heat, while burning of Secale is ameliorated by cold. There is another grand feature of this remedy that makes it again similar to Arsenicum—the cold, clammy sweat all over the body, particularly the limbs. We have restless anxiety, prostration, burning, and cold sweat and clamminess in Arsenicum. Both of these remedies have that state. Now, there are conditions wherein you cannot distinguish these remedies excepting by the aggravations or ameliorations.

In peritonitis, in a state commonly called inflammation of the bowels, Arsenicum and Secale both result in gangrenous conditions. You have a threatened gangrene. You have a horrible burning pain in the abdomen. There is generally the history of a chill and a fever, and the feeble pulse, which are no guides to the remedy at all.

The great pains in the bowels; the restlessness, anxiety, and thirst; vomiting of flakes of blood; bloody mucus discharges from the bowels, thin and watery, and so offensive that it is called cadaverous.

Arsenic has all this state. Secale has all this state.

Both remedies run through with anxiety and prostration and the horrible thirst, and vomiting everything that goes into the stomach, with blood. Hemorrhage from the cavities.

Here you see the individualization. All you have to do is to observe the aggravation from heat or cold. If it is ameliorated by warmth it cannot be Secale. If it is ameliorated by cold, it cannot be Arsenic.

Even in a cold room the Secale patient wants to be uncovered. Though he is covered with a cold sweat, and is clammy, he wants to be uncovered.

And, although he is burning in Arsenic, he wants to be covered up. Heat actually relieves or ameliorates the burning in Arsenic. That is a peculiar symptom of Arsenic.

Now, we often have in the various hemorrhages this burning and this prostration, as a general thing, and Arsenic-hemorrhage is only marked when we have this threatened gangrenous state, and in the typhoid condition of the bowels.

But when it comes to uterine hemorrhages and the passive and protracted hemorrhages of Secale, there we have no relation to Arsenic. It has in this what Arsenic has not.

But it is in this acute condition, such as we find in the gangrenous state, when the patient is right on the line of passing over, if you don't do something quick, he is going to die.

In those complaints in which you have several days to consult your books, we have no similarity to Secale and Arsenic.

In cases where there are uterine complications, where the lady has a continual oozing—a general anemic state—better from cold; shriveled condition, anxiety, restlessness, and thirst—oozing and hemorrhages—shriveled condition and numbness of the limbs; where the hemorrhage has been so marked at first, dwindling away into oozing; that is, protracted; that is, troublesome, thin, watery, black blood that won't coagulate, then we have Secale and no other remedy. There is no other remedy having all those states like Secale.

We have the low hippocratic countenance, as in Arsenic.

Secale doesn't possess the power like China to restore the body to its normal condition. It seems to overcome this shriveled condition. It seems to begin the case where there has been a protracted and tedious hemorrhage.

Where there is a hemorrhage of this kind with all these symptoms, Secale stops it; but the patient doesn't react.

Secale has no tendency to build one up. Now comes the time for China, which is complementary to Secale.

When Secale has stopped the hemorrhage, then follow with China to restore the patient.

Hemorrhage comparisons:

The main symptoms, as we see, show a relation to Arsenic.

Fear of death and anxiety, prostration from anemia of the brain.

It is not related to Aconite in any particular, so we will not compare it with that remedy.

Great nausea, and with the nausea, anxiety, sadness, and melancholy.

Feeling of lightness of the head, mostly in the occiput. This is the result of anemia.

In many ways this brings about a state peculiar to the decline of old age.

You will find many of the complaints of these Secale or smut rye-bread eaters that correspond, coming on in middle life, that correspond to the complaints of old age.

Senile catalepsy has been produced by it in these people who eat this bread containing the smut rye; it is common.

In the nose bleed, blood dark; runs continuously; an oozing—a gradual oozing of thin blood from the nose. In old people or in drinkers, in young women; nose stopped up, yet watery discharges running from it.

Face pale; pinched, pale, earthy looking.

Sunken eyes; blue rings around the eyes. This goes on until hippocratic countenance appears. That we see in very few remedies; like Arsenic, Secale has tingling in the face.

There is a drawing sensation, as if all the tissues of the body were drawing.

In relation to the teeth, bleeding of the gums. The slightest wounds bleed—ooze a long time from a little break of the gum. After tooth is extracted will ooze a long time; not profusely, but a gradual oozing. There seems to be no tendency for it to stop.

Looseness of the teeth. This is a very peculiar and common feature of those who who have eaten this bread containing smut rye—the teeth fall out—that is, within a few months; sinking away of the gums.

Spasm of the tongue, by which the tongue is projected violently forward against the teeth, and if the mouth is open it will thrust out like the tongue of a snake.

Thirst. Unquenchable thirst, burning in all stages of the fever.

Dryness of the soft palate and œsophagus. Thirst.

Burning is characteristic of the remedy in general, and violent thirst.

Painful tingling in the throat and tongue. Tingling of the limbs and fingers. Formications throughout the body from the spinal anemia perhaps. That is produced by the taking of Secale.

Vomiting of black blood, which is very characteristic of Secale.

The continuous nausea is like that of Arsenic.

In Asiatic cholera with collapse—a crawling sensation as from ants; that is the formication in the extremities and in the skin and beneath the skin.

Discharges from the bowels are dark-colored, very fœtid, sometimes an olive-green. They are involuntary and very exhausting, likely to be composed largely of blood.

In the advanced state, when the disease resembles this remedy, if you have suppression of urine, it is perfectly in harmony with Secale.

It has retention of the urine, and it has suppression in most all of these low forms of disease—these forms that correspond with this picture of disease—diseases in which you will see this picture of Secale may have retention, and may have suppression of urine, commonly suppression. There is no urine in the bladder.

Urine is pale, watery, and the characteristic is bloody—bloody in old people, bloody in young people.

Black blood from the bladder, thick; still it does not coagulate.

Particularly does Secale act upon the kidneys, as much so as Belladonna.

There is great similarity between Secale and Belladonna.

Belladonna acts more violently. It produces contraction of the calibre of the blood-vessels—turgescence.

Secale produces a continued anemia that lasts much longer, and the relaxlation following it occurs at about the time of the breaking down of the blood corpuscles, hence we have an anemia of an organic kind, as well as the anemia common to both of these remedies.

Female sexual organs. Very important and characteristic.

Menses too profuse and last too long, with tearing and cutting colic; cold extremities.

Menstrual blood thin and black—very peculiar; don't forget that.

Uterine hemorrhage from the slightest motion. That is not peculiar to this remedy; it is thin fluid of a disgusting smell. Menstrual flow is very fœtid; it is strong and pungent, and the hemorrhage has that peculiar odor of atonic hemorrhage during the critical age.

Uterine ulcer feels as if burnt.

Discharges putrid, and fluid blood; burning after the flow; thin fluid blood with exhaustion; restlessness; anxiety; thirst; continued passive hemorrhage.

The leucorrhœa has this peculiar smell; it is brownish and offensive looking. Very offensive smell.

You don't see anything like rapidly flowing or bright red blood, and very little gush of blood, attended with faintness. No overpowering nausea; that is Ipecac, and Ipecac only.

The contracted uterus fills up with clots of blood coming away as large as a fist, followed by a profuse hemorrhage, with lancinating pains going backward; you dam it up, or by some means or other it stops; the uterus fills up with a clot of blood; finally contraction comes on and it gives way and the pains run from before backward. You don't see that in Ergot, for it is not there. That is Sabina.

You have perhaps an enormously distended uterus, not so much as in pregnancy. There seems to be general weakness, because of this exhaustion; because of this burning; because of this thirst; but there is simply inertia of the uterus; it does not contract at all; it simply fills up with blood. You can stick your three fingers into the uterus, but even then there is no contraction; she has been tamponed, perhaps, and the uterus fills up with blood; finally, the clot breaks loose, and it is followed by a painless state, but not by the burning, by the anxiety, nor by the horrible prostration and thirst that you find in Ergot.

This inertia, this general state of relaxation of the uterus, is peculiar to Caulophyllum.

Suppose you have another picture of this kind—a continuous protracted oozing of blood, bright arterial blood; uterus is contracting, but still there is an oozing of bright red blood. With the continued oozing you have no thirst; no fever; the patient says if this oozing of blood would only stop, she would be perfectly well. You have no symptoms for Secale. You think you will have to give a big dose to control that, because you haven't any symptoms to go on, and the patient would be well if it was not for this oozing hemorrhage. But that doesn't relieve this bright, red blood that is oozing continuously. The remedy is Millefolium.

A very celebrated man in the United States had an oozing from the rectum, caused by a fall; local applications had failed to stop it. He was apparently well. There seemed very little the matter with him, except that continuous oozing from the rectum. After months of this annoying oozing of bright red blood from the rectum, his good wife wrote to Dr. Lippe at Philadelphia, and the good Lippe sent him one dose of Millefolium, because he couldn't send anything else. That was enough, and the oozing stopped very promptly.

Suppose you had a continuous oozing of dark blood without any symptoms, without any burning, without this restlessness, without this exhaustion, without these terrible shriveling symptoms, without this aggravation from heat and amelioration from cold, just simply an oozing; that would be Hamamelis, which has also a flow of bright blood.

Suppose you have this relaxed condition again; you introduce a finger into the vagina, and as you bring it out it is covered with a thick coating, like New Orleans molasses, only not so sticky, that is so peculiar that I am hardly able to describe it; in addition, too, there is the burning of Secale; there is not that nausea, but there is that thirstlessness. Now Ustilago has that state, that peculiar slimy, dark, uncoagulable blood. It is thick.

In Belladonna the blood feels hot and the flow is passive. It comes away in gushes and feels hot to the parts—intensely hot. She is worse from the least jar. If a lady tells you that her discharges have this hot feeling, either her menstrual flow or the hemorrhage, as you step up to the bed, it is a very convenient thing to do, to give your hand or leg a jar against it; if it is a Belladonna case it will be aggravated by the least jar; it is aggravated from the jar, she is annoyed, it is painful, she is sore, there is some congestion and tenderness in the uterus. That peculiar jar of the bed, she don't want you to do it again, and her countenance is very threatening. When I see from the general symptoms and the condition of the patient that Belladonna is indicated, I then give the bed a rap; if she doesn't pay any attention to it, there is no Belladonna case.

Suppose there is oozing of blood, which does not coagulate, and the remedy seems clearly indicated, but it doesn't act. You will be apt to say, I have tried Homœopathy for the symptoms and it doesn't act. I will give a big dose of Ergot and stop it that way. But a big dose won't always stop it, no matter how big the dose.

You must give Sulphur if Secale fails to act. It is an intercurrent.

If your hemorrhage stops, as it often does under the Sulphur itself, you needn't feel that Sulphur has cured. The Sulphur has simply permitted the indicated remedy to act—the cure by Ergot has been hastened by Sulphur. You sometimes have to change from Ergot to Sulphur; after having given Sulphur you sometimes have to change, and then in about twenty-four hours you can give the indicated remedy again.

